# Determining Cut-Off Points for the Dental Fear Survey

**DOI:** 10.1155/2015/983564

**Published:** 2015-09-29

**Authors:** Maurício Antônio Oliveira, Cristiane Baccin Bendo, Saul Martins Paiva, Miriam Pimenta Vale, Júnia Maria Serra-Negra

**Affiliations:** Department of Pediatric Dentistry and Orthodontics, School of Dentistry, Universidade Federal de Minas Gerais, Avenida Presidente Antônio Carlos 6627, 31270-901 Belo Horizonte, MG, Brazil

## Abstract

*Objective*. To determine a high fear cut-off point score for the Dental Fear Survey (DFS) using a single-item self-report questionnaire. *Methods*. The DFS, a 20-item questionnaire assessing fear of dental treatment, was completed by 1,256 participants with a mean age of 22.3 years (SD = 5.1). Another self-report questionnaire was used to collect data on previous dental experiences. A high fear cut-off point score was determined by calculating the receiver operating characteristic (ROC) curve for the DFS. Descriptive statistics and multinomial logistic regression were calculated; a significance level of *p* < 0.05 was used for all tests. *Results*. The ROC curve indicated that a DFS score ≥53 corresponds to a sensitivity of 88.9% and a specificity of 92.5%. Most participants (*n* = 895; 71.5%) reported no fear of going to the dentist. There was significant association between DFS score and fear assessed with the question “Are you fearful of going to the dentist?” (*p* < 0.001). *Conclusion*. A cut-off point of 53 on the DFS total score represents the best compromise between sensitivity and specificity and can be used to predict high dental fear.

## 1. Introduction

Both the theoretical aspects and practical consequences of dental fear have been investigated over the past 50 years [1, 2]. Dental fear may originate in negative dental experience during childhood [3–11]; behavioural problems during treatment may be influenced by the dentist's behaviour [7, 12, 13]; patient personality traits and other specific fears may also play a role [14–16].

Studies of dental fear play an important role in predicting behavioural problems in dentally fearful individuals that can result in avoidance or irregular visits [4–6, 8, 12, 16–19]. The reported prevalence of dental fear in adults ranges from 3.3% to 31.8% [6, 8, 12, 17, 18, 20–25]; this wide range may reflect cultural differences, previous dental experience, or use of different measures and different cut-off points for defining dental fear [1, 26].

Generally, dental fear is measured according to cut-off points on validated self-reported scales [27]. Most studies have used the mean sum score (±SD) or median score on the DFS as a cut-off point [6, 28–33]. Use of these measures of central tendency can bias conclusions, as the researchers predetermine the percentage of the population who will be categorised as dentally fearful. Different cut-off points or scales in the same population can result in diverse interpretations of assessing dental fear [27].

There is considerable variability in the instruments used to determine dental fear and there is no recognised gold standard [27]. Several cut-off points have been applied to the DFS to indicate high dental fear [13, 20, 21, 23, 26, 28, 32, 34, 35]. A cut-off point is a point on a continuous measure that acts as a categorical boundary, ideally providing an intuitive interpretation of scores above and below that point [27, 36]. It is important to use a quantitative diagnostic test to determine the cut-off point on a continuous scale that would best enable dentists to identify fearful individuals. The main objective of this study was to define a “high fear” cut-off point for the DFS by optimising the sensitivity and specificity of a diagnostic test (the DFS) according to the response to the direct question from a self-report questionnaire on dental experiences: “Are you fearful of going to the dentist?”

## 2. Materials and Methods

### 2.1. Population and Settings

The data for this study were derived from a preexisting field study database [11, 37]. The objective of this study was to define cut-off points for the DFS. A large convenience sample of undergraduates from health (dentistry), hard (mathematics), and soft (psychology) sciences were recruited from the Universidade Federal de Minas Gerais (UFMG) between August and December 2010. All the 1,565 students enrolled in these three undergraduate courses were invited to participate in the study. Undergraduates were residents of the city of Belo Horizonte, Minas Gerais, Brazil. Following authorisation by the Human Research Ethics Committee of UFMG (ethics approval for the 0201.0.203.000-10 protocol), written informed consent was obtained from 1,256 participants, who completed two self-report questionnaires: the Brazilian version of the DFS and a pretested questionnaire.

Potential participants were approached during lecture classes and asked if they were prepared to participate in the study.

### 2.2. Measure

The Brazilian version of the DFS was used to collect data [28]. This is a 20-item questionnaire relating to dental treatment, comprising three subscales: Avoidance (eight items), Physiological Arousal (five items), and Fears of Specific Stimuli/Situations (seven items). Each item has a five-point response rating ranging from “not at all” (1) to “very much” (5). Avoidance scores range from 8 to 40; Physiological Arousal scores range from 5 to 25; and Fears of Specific Stimuli/Situations scores range from 7 to 35. Higher scores indicate greater dental fear [28]. The DFS was developed to assess dental fear through questions about behavioural, physiological, and cognitive responses to specific dental treatment procedures [12]. Another questionnaire was used to collect data about previous dental experiences. This questionnaire is a 17-item self-report and was developed and pretested by the authors' research.

The two questionnaires were administered in a pilot study of 80 students from the three courses on two occasions, separated by an interval of two weeks. These students did not participate in the main study. The intraclass correlation coefficient (ICC) for test-retest reliability of the DFS for mathematics, dentistry, and psychology undergraduate students was 0.969 (95% CI: 0.945–0.986), 0.968 (95% CI: 0.953–0.980), and 0.949 (95% CI: 0.911–0.977), respectively. Additional information on dental fear was collected using a single question from the second questionnaire “Are you fearful of going to the dentist?” with four response options, “not fearful”; “a little fearful”; “very fearful”; and “extremely fearful.” Cohen's kappa statistic for test-retest to the question “Are you fearful of going to the dentist?” showed a good degree of agreement (*K* = 0.680, *p* < 0.001). This question was used only to define the DFS cut-off points according to the methodology of the ROC curve. The pilot study indicated that changes to the proposed methodology were not necessary.

### 2.3. Variables

Dental fear was the main outcome variable; it was indexed by total DFS score and treated as a continuous variable. The distribution of DFS scores was analysed to define two cut-off points using the receiver operating characteristic (ROC) curve, thus creating three dental fear categories: a “not fearful” group, a “low fearfulness” group, and a “highly fearful” group. The independent variables were gender, negative dental experiences in childhood, and the response to the question “Are you fearful of going to the dentist?”

### 2.4. Statistical Analysis

Statistical analysis was conducted using descriptive statistics, the ROC curve, the Kruskal-Wallis test, and multinomial logistic regression; the significance level was set at 5% for all tests. The Statistical Package for the Social Sciences, version 22.0 for Windows (SPSS Inc., Chicago, IL), was used to conduct statistical analysis.

To calculate the ROC curve responses to the question “Are you fearful of going to the dentist?” it was coded as a binary variable (“not fearful” and “fearful”), to distinguish between individuals without fear and those who were fearful. A dichotomisation was performed to define a cut-off point to identify individuals who were highly fearful. Participants who had responded “not fearful” were assigned to the “not fearful” category; all other participants were assigned to the “fearful” category (“a little fearful”; “very fearful”; and “extremely fearful”). Then a different binary categorisation was used: “highly fearful” and “not fearful/low fearfulness.” The “highly fearful” category was made up of participants who indicated that they were “very fearful” or “extremely fearful,” whilst participants who had responded that they were “not fearful” or “a little fearful” were assigned to the “not fearful/low fearfulness” category. This dichotomisation was important for determining the values of DFS scores that identify individuals with dental fear.

The ROC curve was used to determine DFS cut-off points based on self-reported fear of going to the dentist. The ROC curve is a graphical plot of sensitivity against 1 − specificity at various discrimination cut-off points. The best cut-off point is the one that represents the best compromise between sensitivity and specificity.

The normality of the data was assessed using the Kolmogorov-Smirnov test; this test indicated that the data were not normally distributed. A Kruskal-Wallis test was used to associate DFS scores to responses to the question “Are you fearful of going to the dentist?”

Multinomial logistic regression was used to assess whether the independent variables (gender and negative dental experience in childhood) were related to fear of going to the dentist in terms of the three categories defined by DFS scores. Observed values were the numbers in the fear categories (“not fearful,” “low fearfulness,” or “highly fearful”) defined in terms of DFS cut-off points. Predicted values were the numbers in these respective groups of fear predicted by the logistic regression model.

## 3. Results

### 3.1. Characteristics of the Participants

The two self-report questionnaires were completed by 1,256 dentistry, psychology, and mathematics undergraduates, representing a response rate of 80.25%. The age of participants ranged from 18 to 65 years, with a mean of 22.3 years (SD = 5.1); 37.1% were men and 62.9% were women. In response to the question “Are you fearful of going to the dentist?” most participants (71.5%) reported that they had no fear of going to the dentist and only 3.6% indicated that they had a high level of fear (“very fearful” or “extremely fearful”). A statistically significant relationship was found between total DFS score and directly self-reported fear; that is, participants with a lower DFS score were more likely to belong to the “not fearful” group whereas participants with a higher DFS score were more likely to belong to the “highly fearful” group (*p* < 0.001) (Table 1).

### 3.2. Cut-Off Points Determination

**Figure 1:**
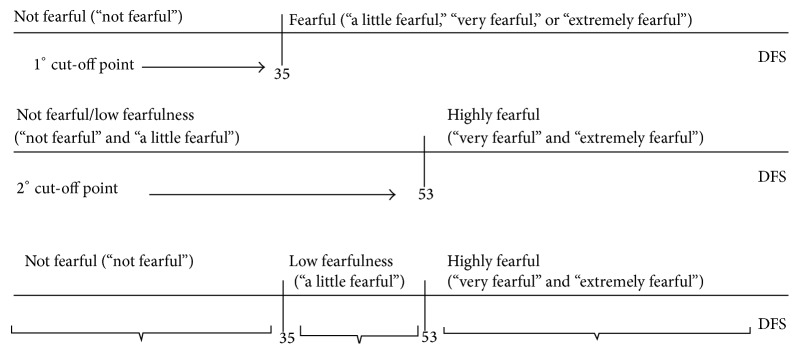
Determination of DFS cut-off points on the basis of the receiver operating characteristic (ROC) curve for responses to the question “Are you fearful of going to the dentist?” Not fearful (DFS score ≤ 35), a little fearful (36 ≤ DFS score ≤52), and highly fearful (DFS score ≥ 53).

DFS cut-off points were based on responses to the question “Are you fearful of going to the dentist?” Two cut-off points were identified from the ROC curve and used to classify DFS respondents into three dental fear groups: “not fearful” (DFS score ≤ 35), “a little fearful” (36 ≤ DFS score ≤ 52) and “highly fearful” (DFS score ≥ 53) (Figure 1).

### 3.3. Sensitivity and Specificity

**Figure 2:**
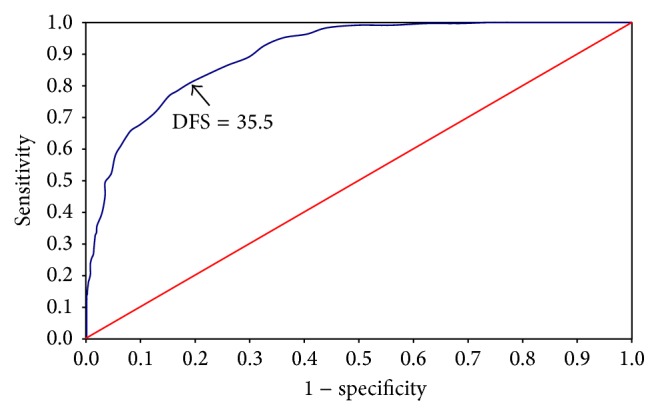
Receiver operating characteristic (ROC) curve for the Dental Fear Survey (DFS). Responses to the question “Are you fearful of going to the dentist?” were assigned to binary categories, “not fearful” or “fearful.” A DFS score of 35.5, representing the best compromise between sensitivity (0.81) and specificity (0.81), was chosen as the cut-off point for “fearful.” Area under the curve (AUC) = 0.903; *p* < 0.001.

**Figure 3:**
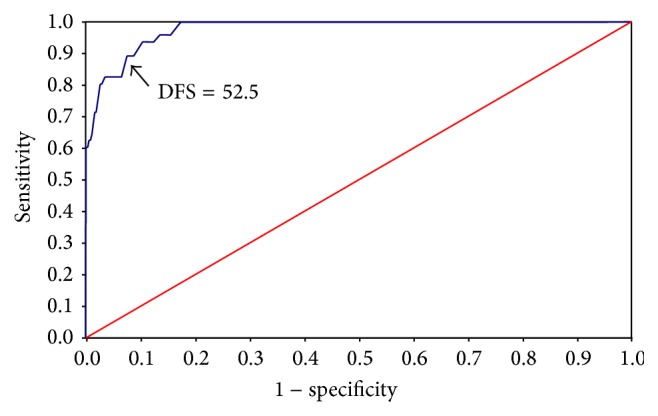
Receiver operating characteristic (ROC) curve using the Dental Fear Survey (DFS). Responses to the question “Are you fearful of going to the dentist?” were assigned to binary categories, “not fearful/low fearfulness” (“not fearful” and “a little fearful”) and “highly fearful” (“very fearful” and “extremely fearful”). A DFS score of 52.5, representing the best compromise between sensitivity (0.89) and specificity (0.92), was chosen as the cut-off point for “highly fearful.” Area under the curve (AUC) = 0.977; *p* < 0.001.

The ROC curve was used to estimate cut-off point scores that reflected the binary “not fearful” and “fearful” classification of responses to the single direct question about fear of going to the dentist. The area under the curve (AUC) was 0.903 (*p* < 0.001), and the DFS cut-off point which gave the best compromise between sensitivity (81.0%) and specificity (81.0%) was 36 points (Figure 2; Table 2). The ROC curve for the binary categorisation “not fearful/low fearfulness” or “highly fearful” had AUC of 0.977 (*p* < 0.001) and DFS cut-off point of 53 points, corresponding to a sensitivity of 88.9% and a specificity of 92.5% (Figure 3; Table 2). Table 2 indicates the decrease in sensitivity and increase in specificity above 52.5 points. The percentage of false negatives (individuals mistakenly identified as being without dental fear) was reduced markedly by raising the DFS cut-off point above 52.5 points (Table 2).

### 3.4. Associated Factors

A multinomial logistic regression controlling for undergraduate course showed that the two independent variables, gender and negative dental experiences in childhood, were associated with dental fear. High dental fear was more prevalent among females (OR = 1.63; 95% CI = 1.09–2.44). Individuals who had had negative dental experiences in childhood were more likely to report high dental fear in adulthood (OR = 5.09; 95% CI: 3.47–7.44) (Table 3).

### 3.5. Dental Fear among Study Groups Based on Adjusted Model

The multinomial logistic regression model was used to assign individuals to one of the three DFS groups (“not fearful,” “low fearfulness,” or “highly fearful”) to get an estimated or predicted fear categorisation. The logistic regression model was evaluated by comparing the “observed” and “predicted” DFS categories (Table 4). The adjusted model showed that almost all individuals assigned to the “not fearful” group (95.2%) were also assigned to the “not fearful” group by the model, whereas only 3.4% and 29.2%, respectively, of the “low fearfulness” and “highly fearful” groups were correctly assigned by the model (Table 4).

## 4. Discussion

Different cut-off points have been used to produce categorical variables from continuous scales and this makes it difficult to compare studies [8]. Several previous studies have used empirical criteria to define DFS cut-off points for high fear with values ranging from 45 to 60 [13, 20, 26, 28, 32, 34, 35]. A consensus about cut-off points for dental fear is critical to comparative research [27, 36]. This present study makes an important contribution to the knowledge, as responses to a single-item diagnostic test were plotted as ROC curves and used to determine fear cut-off points. Analysis of the ROC curves revealed a significant association between fear categories defined in terms of DFS cut-off points and responses to a direct question about fear of going to the dentist; that is, the “not fearful/low fearfulness” DFS category corresponded to being “not fearful” or “a little fearful” of going to the dentist, whilst the “highly fearful” DFS category corresponded to being “very fearful” or “extremely fearful” of going to the dentist.

Although the range of possible DFS scores is 20 to 100, this study showed that the cut-off point for “highly fearful” was only 53. The high sensitivity (88.9%) and specificity (92.5%) at this cut-off point indicated good agreement between categorisation on the basis of DFS score using this cut-off point and responses to the direct question “Are you fearful of going to the dentist?” In this present study using a cut-off point of 53 provided a reliable classification; a similar cut-off point (55) was defined by Firat et al. [23] in a study which included the assessment of sensitivity and specificity of the DFS, although with older participants drawn from a different population. Accurate and reliable assessment of a patient's fear is important as it influences the approach to dental treatment [36].

The multinomial logistic regression model assigned 1,094 individuals to the “not fearful” group; however, the observed responses of 230 (21.0%) and 108 (9.9%) of these individuals placed them in the “low fearfulness” and “highly fearful” categories, respectively (Table 4). By way of explanation roughly one in 10 individuals in the “not fearful” DFS group actually reported a high level of fear in response to the direct question. These individuals might have been underestimating their fear in specific situations at the dentist. Fifty-three (42.1%) and 24 (19.0%) of the 126 individuals assigned to the “highly fearful” group by the logistic regression model were categorised as having a “low fearfulness” or being “not fearful,” respectively, on the basis of their responses to the direct question. This suggests that two out of 10 individuals classified as “highly fearful” based on the DFS do not need special attention to manage fear during dental care (Table 4). Individuals who are fearful of particular stimuli or dental treatment situations may not have an overall fear of going to the dentist. This discordance between the DFS and a direct question about fear does not mean that the DFS should be considered inaccurate [38]; in this instance, it would be better to overestimate rather than underestimate fear.

Gender contributed to variance in dental fear. Women had more dental fear than men when the three categories of fear were compared; however when dental fear was coded as a binary variable (“highly fearful”; “low fearfulness”) there was no effect of gender (*p* = 0.520). As in many previous studies [5–11], negative dental experiences in childhood were associated with high dental fear in adulthood (*p* < 0.001). In this study defining fear cut-off points for the DFS increased the clinical interpretability of the scale and provided evidence of an association between risk factors and high dental fear.

The limitations of the study should be taken into account when interpreting the results. Self-report instruments which assess past dental experiences may be subject to recall bias, respondents may differ in how they interpret the various statements about fear, and evaluations may be influenced by the salience of particular dental experiences [39, 40]. In addition, the DFS does not assess all factors related to dental fear, for example, personality factors. The sample consisted of young undergraduates, with an average age of 22.3 years (SD = 5.1), who probably had little negative experience of dental treatment and this may have affected the results. Prevalence of dental fear tends to be lowest in young adults [27]. The sample size was large, but because the sample was composed exclusively of undergraduates, the external validity of the findings is open to question. Further studies are necessary to confirm that these findings could be generalised to the wider population.

These results are directly relevant to dental research and practice. Previous study with Brazilian undergraduates demonstrated a mean score for DFS of 35.2 ± 13.10 (11). Studies conducted with adults from other countries presented slightly different results, such as mean scores of DFS in Greece (39.8 ± 17.5) [29], Japan (37.4 ± 14.1) [31], Germany (42.7 ± 17.6) [32], and Turkey (36.1 ± 16.2) [33]. Given the population differences, these results demonstrated the importance of using valid instruments that capture the cultural differences across countries. It is fundamental to understand the cultural differences in measuring a construct as dental fear. Because of that, the cross-cultural adaptation and validation process are necessary to provide an instrument with face, content, and construct validity for each culture.

Dental fear has been recognised as an important problem and is often a barrier to successful dental treatment [26, 36, 41–43]. Dentists should listen to their patients when it comes to negative experiences of treatment and fear in a clinical setting. A “highly fearful” categorisation should be identified so that it reflects the patient's behaviour and provides a meaningful indicator of fear which can be used to determine the appropriate approach to dental treatment.

## 5. Conclusions

The results suggest that ROC curves can be used to identify cut-off point for dental fear categories based on optimising sensitivity and specificity. A DFS score of 53 represented the best compromise between sensitivity and specificity and was selected as the cut-off point for high dental fear. There was a significant association between responses to a direct question about dental fear “Are you fearful of going to the dentist?” and categories based on DFS score using this cut-off point. Individuals with a DFS score ≥ 53 must be treated as highly fearful and precautions must be taken to avoid behavioural problems during dental treatment.

## Figures and Tables

**Table 1:** Total DFS scores for participants grouped according to response to the direct question “Are you fearful of going to the dentist?” (*n* = 1,252).

Groups of fear	DFS total scores				
	*N*	Minimum	Maximum	Median	Mean (SD)
Not fearful	895	20.0	63.0	27.0	29.4 (8.0)
A little fearful	312	24.0	76.0	45.0	45.2 (10.4)
Very fearful	27	45.0	100.0	76.0	75.0 (12.3)
Extremely fearful	18	48.0	89.0	62.0	63.5 (12.2)

Kruskal-Wallis test (*p* < 0.001).

SD = Standard Deviation.

Conclusion = not fearful < a little fearful < highly fearful (very fearful and extremely fearful).

**Table 2:** Determination of DFS cut-off points sensitivity and specificity based on responses to the question “Are you fearful of going to the dentist?”

Cut-off point (total DFS score)	**Not fearful** *versus* **Fearful** (“a little fearful,” “very fearful,” and “extremely fearful”)		**Low fearfulness** (“not fearful” and “a little fearful”) *versus* **Highly fearful** (“very fearful” and “extremely fearful”)	
	Sensitivity	Specificity	Sensitivity	Specificity
20.5	1.000	0.102	1.000	0.075
25.5	0.997	0.375	1.000	0.279
30.5	0.952	0.638	1.000	0.487
**35.5**	**0.810**	**0.810**	1.000	0.657
40.5	0.678	0.902	1.000	0.764
45.5	0.527	0.954	0.956	0.846
50.5	0.359	0.981	0.889	0.913
**52.5**	0.328	0.984	**0.889**	**0.925**
55.5	0.255	0.991	0.822	0.949
60.5	0.182	0.997	0.800	0.973
65.5	0.106	1.000	0.622	0.992
70.5	0.073	1.000	0.556	0.999
75.5	0.050	1.000	0.378	0.999

A DFS score of 35.5, representing the best compromise between sensitivity (0.81) and specificity (0.81), was chosen as the cut-off point for **dental fear**. A DFS score of 52.5, representing the best compromise between sensitivity (0.89) and specificity (0.92), was chosen as the cut-off point for **highly fearful category**.

Cut-off points are given in bold.

**Table 3:** Multinomial logistic regression model of independent variables for fear categories based on DFS cut-off points.

Variables	Not fearful/highly fearful		Low fearfulness/highly fearful	
	OR/95% CI	*p* value	OR/95% CI	*p* value
Intercept		**<0.001**		**0.001**
Gender	1.63 (1.09–2.44)	**0.019**	1.15 (0.74–1.79)	0.520
Negative dental experience in childhood	5.09 (3.47–7.44)	**<0.001**	2.09 (1.41–3.11)	**<0.001**

Model adjusted for undergraduate course.

Reference = high fearful category.

Statistically significant results are given in bold.

OR = Odds Ratio.

CI = Confidence Interval.

*p* value = probability value.

**Table 4:** Multinomial logistic regression model adjusted for predicting the fearfulness of going to the dentist comparing observed values (responses to direct question) with predicted values (based on DFS scores and cut-off points) (*n* = 1,255).

Observed value	Predicted value				Correct classification (%)
	Not fearful	Low fearfulness	Highly fearful	Total	
Not fearful	***756***	14	24	794	95.2
Low fearfulness	230	***10***	53	293	3.4
Highly fearful	108	11	***49***	168	29.2
Total	**1,094**	**35**	**126**	1,255	64.9
	69.1%	28.6%	38.9%		
